# Alteration of the α_1_β_2_/α_2_β_1_ subunit interface contributes to the increased hemoglobin-oxygen affinity of high-altitude deer mice

**DOI:** 10.1371/journal.pone.0174921

**Published:** 2017-03-31

**Authors:** Noriko Inoguchi, Nobuhiro Mizuno, Seiki Baba, Takashi Kumasaka, Chandrasekhar Natarajan, Jay F. Storz, Hideaki Moriyama

**Affiliations:** 1 School of Biological Sciences, University of Nebraska, Lincoln, Nebraska, United States of America; 2 Protein Crystal Analysis Division, Japan Synchrotron Radiation Research Institute, Sayo, Japan; Russian Academy of Medical Sciences, RUSSIAN FEDERATION

## Abstract

**Background:**

Deer mice (*Peromyscus maniculatus*) that are native to high altitudes in the Rocky Mountains have evolved hemoglobins with an increased oxygen-binding affinity relative to those of lowland conspecifics. To elucidate the molecular mechanisms responsible for the evolved increase in hemoglobin-oxygen affinity, the crystal structure of the highland hemoglobin variant was solved and compared with the previously reported structure for the lowland variant.

**Results:**

Highland hemoglobin yielded at least two crystal types, in which the longest axes were 507 and 230 Å. Using the smaller unit cell crystal, the structure was solved at 2.2 Å resolution. The asymmetric unit contained two tetrameric hemoglobin molecules.

**Conclusions:**

The analyses revealed that αPro50 in the highland hemoglobin variant promoted a stable interaction between αHis45 and heme that was not seen in the αHis50 lowland variant. The αPro50 mutation also altered the nature of atomic contacts at the α_1_β_2_/α_2_β_1_ intersubunit interfaces. These results demonstrate how affinity-altering changes in intersubunit interactions can be produced by mutations at structurally remote sites.

## Introduction

Hemoglobin is a heterotetrameric protein consisting of two α subunits and two β subunits, and each subunit has an iron-centered heme that reversibly binds oxygen [1]. Hemoglobin transports heme-bound oxygen from the lungs to all of the tissues in the body via oxygenation-linked shifts in the conformational equilibrium between the tense state (T state; deoxygenated) and the relaxed state (R state; oxygenated) [1–3]. This structural transition is controlled allosterically, as the binding of oxygen to one subunit affects the oxygen-affinity of the other subunits in the same tetrameric assembly [3]. Therefore, studies of structure-function relationships in hemoglobin have focused on heme-ligand binding at the active site [4, 5] as well as the oxygenation-linked conformational changes between the R and the T states [6–8]. Recent studies suggest the presence of several intermediate states between structural transitions [9–11].

**Fig 1:**
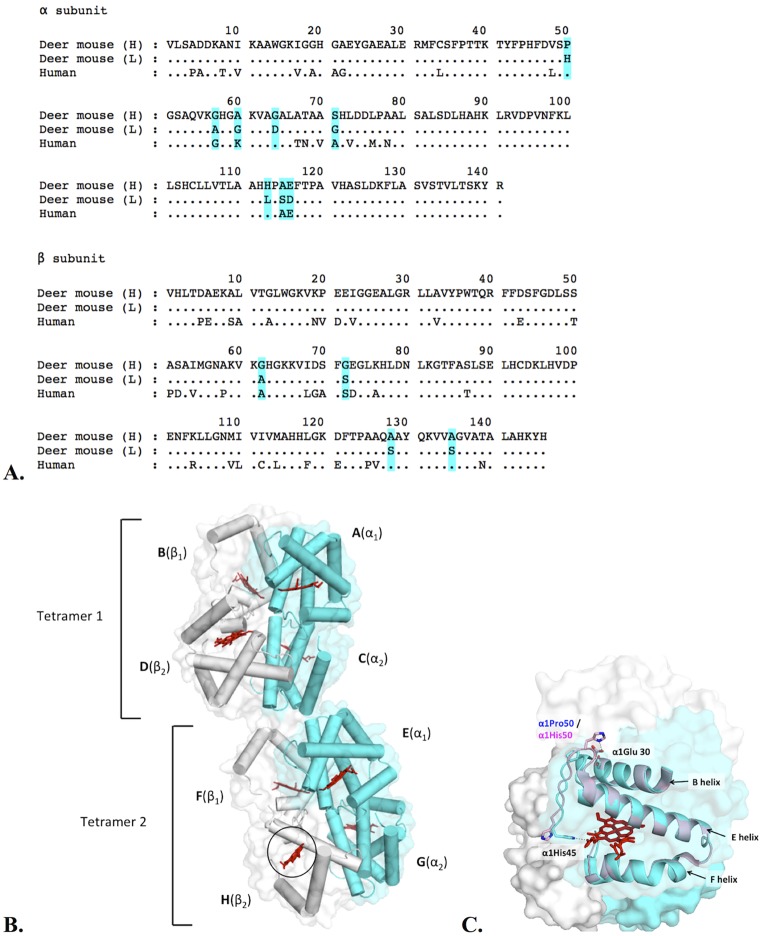
Sequence comparisons and differences in the α subunit main chain coordinates between highland and lowland hemoglobin. (A) Sequence alignment of deer mouse highland (H) and lowland (L) hemoglobin. The substitutions are highlighted in blue. The sequences were obtained from the deposited structures as follows: highland deer mouse hemoglobin (PDB ID: 5KER), lowland deer mouse hemoglobin (4H2L), human hemoglobin (2DN1). (B) Orientation of two tetrameric hemoglobin molecules in an asymmetric unit. The α subunits are colored in cyan, and the β subunits are colored in gray. Heme in each subunit is shown as a red stick model, and the active site without ligand assignment is indicated with a black circle. (C) Structural difference in main chain folding with an emphasis on αHis45 coordinates. Tetrameric highland hemoglobin (5KER; cyan) and lowland hemoglobin (4H2L, pink) are superimposed by PyMOL. The residues around the active site and the nearby loop region are shown in the cartoon model. Key residues and helices containing these residues in the α subunit (chain A) are shown in the stick model and the cartoon model.

When air-breathing vertebrates are exposed to environmental hypoxia, an increase in hemoglobin-oxygen affinity can compensate for the reduced partial pressure of oxygen in inspired air by safeguarding arterial oxygen saturation [12–14]. To investigate the structural basis of evolved changes in hemoglobin-oxygen affinity, we chose to focus on functionally well-characterized hemoglobin variants of the deer mouse, *Peromyscus maniculatus*. Populations of deer mice that are native to high-altitudes in the Rocky Mountains have evolved a genetically based increase in hemoglobin-oxygen affinity relative to lowland conspecifics in the prairie grassland. The hemoglobin of the highland mice has a higher intrinsic oxygen-affinity, and this affinity difference is further accentuated in the presence of chloride ions and 2,3-disphophoglycrate (DPG)[15–19]. The most common hemoglobin variants of high- and low-altitude deer mice are distinguished by a total of 12 amino acid substitutions–eight in the in the α subunit and four in the β subunit (Fig 1A). The oxygenation properties of these alternative variants are well-characterized [15–19], but the structural basis of the observed differences in oxygen affinity has not been fully elucidated.

Previously, we solved the 3D structure of the lowland hemoglobin variant of deer mice (PDB ID: 4H2L), and structural comparisons with human hemoglobin showed that αHis45 is oriented toward the solvent in this lowland variant, whereas αHis45 in human hemoglobin interacts with heme [20]. This unique αHis45 configuration was also observed in horse hemoglobin crystallized with 2-[4-(3,5-dichlorophenylureido) phenoxy]-2-methylpropionic acid (L35) (PDB ID: 2D5X) [21].

We solved the crystal structure of the highland hemoglobin variant to gain further structural insights into the molecular mechanism responsible for the evolved increase in oxygen-binding affinity. A comparative analysis of the highland and lowland deer mouse hemoglobin variants revealed that main chain displacements were present between α41 and α54, the loop region near heme, which resulted from a α50His→Pro mutation in the highland variant. The difference was also highlighted by the interaction between αHis45 and heme in the highland hemoglobin variant. In addition, the highland hemoglobin variant had a reduced intersubunit contact surface, particularly at the α_1_β_2_/α_2_β_1_ switch region.

These analyses suggest that the loose intersubunit interfaces in the highland hemoglobin molecule confers a flexible structure that shifts the allosteric equilibrium towards the R state by lowering the free energy of the R↔T transition, thereby increasing oxygen affinity.

## Materials and methods

This study was carried out in accordance with the recommendations in the Guidelines-Research involving Recombinant or Synthetic Nucleic Acid Molecules of the National Institutes of Health. The protocol was approved by the Institutional Biosafety Committee of the University of Nebraska-Lincoln (Protocol ID: 174).

### Purification and crystallization of recombinant highland hemoglobin

Recombinant highland deer mouse hemoglobin in the carbon monoxide format was produced as previously described [20, 22]. Purified highland hemoglobin was crystalized with the vapor diffusion method using 26% (w/v) PEG 3350 in 50 mM sodium/potassium phosphate buffer, pH 8.0, at 299 K in the airtight vial purged with carbon monoxide (S1 Fig). We dissolved the crystals 65 days after they were formed, and UV-visible spectroscopy revealed the spectrum expected for carboxy hemoglobin. Diffractable crystals were obtained in fiber clusters with 5 mM glutathione, 0.1% (v/v) 2,4-pentanediol and 7 mM calcium chloride (S1 Fig). The lowland hemoglobin variant was crystalized under the same conditions as the highland variant with the exception that 28% (w/v) PEG 3350 was used; however, the same fibrous format was not observed. Crystals of lowland hemoglobin were produced only with 14 mM calcium chloride without glutathione and 2,4-pentanediol [20].

### Preliminary X-ray diffraction analysis

At least two types of crystals were grown. However, in both cases, the crystals were fragile and had a high mosaic spread in the diffraction images. Multiple attempts were required to obtain an interpretable diffraction image by HKL2000 (HKL Research, Charlottesville, VA, USA) [23]. The first crystal (type F) was subjected to the diffraction experiment at BL41XU at the SPring-8. The type F crystal diffracted up to a 3.0 Å resolution, and the crystal belonged to the space group *C*222/*C*222_1_ with the unit cell dimensions *a* = 53, *b* = 91 and *c* = 507 Å. The second crystal (type S) was analyzed at the 14-BM-C, BioCARS of the Advanced Photon Source (APS) at Argonne National Laboratory. The type S crystal diffracted beyond 2.2 Å resolution and belonged to the space group *P*2_1_ with the unit cell dimensions *a* = 53.18, *b* = 229.62, *c* = 53.29 Å and β = 119.12° (Table 1). Considering the compactness of the unit cell, we solved the structure of highland hemoglobin using the type S crystal.

**Table 1:** Data collection and refinement statistics of the type S crystal.

**Data collection**	
Wavelength (Å)	0.978
Resolution range (Å)	29.56–2.202 (2.281–2.202)
Space group	*P*2_1_
Unit cell (Å, °)	53.18 229.62 53.29, 90 119.12 90
Total reflections	65478 (4715)
Unique reflections	39866 (3273)
Multiplicity	1.6 (1.4)
Completeness (%)	0.71 (0.59)
Mean I/σ (I)	9.26 (3.49)
Wilson B-factor	22.28
R-merge	0.08958 (0.2582)
CC_1/2_	0.983 (0.665)
**Refinement**	
R-work	0.1931 (0.3292)
R-free	0.2017 (0.3579)
CC (work)	0.952 (0.635)
CC (free)	0.949 (0.597)
macromolecules	8604
ligands	344
protein residues	1144
RMS (bonds)	0.007
RMS (angles)	1.12
Ramachandran favored (%)	96
Ramachandran allowed (%)	3.5
Ramachandran outliers (%)	0
Rotamer outliers (%)	1.2
Clashscore	7.24
Average B-factor	28.98
macromolecules	28.87
ligands	27.00
solvent	31.33

Statistics for the highest-resolution shell are shown in parentheses.

### Data collection and structure refinement

The diffraction data for the type S crystal were collected from a flash frozen single crystal under the cold stream at 90 K using 10% (v/v) glycerol as the cryo-protectant. Diffraction images were recorded by the ADSC Q315 detector at the 14-BM-C using the X-ray wavelength 0.978 Å at 14-B-C, APS BioCARS. We used the oscillation method with a swing width of 1°. We collected two data sets to cover 180° of the spindle for each using κ angles of 0° and 30°. During data collection, the crystal suffered immense radiation damage. After iterative image data processing using HKL2000 [23], only the first dataset with κ = 0° was usable, in which only 128 images out of 180 images were interpreted. The collected diffraction data exhibited anisotropy, limiting data collection beyond 3.0 Å resolution along the L-axis. This resulted in 71% completeness, but we were still able to achieve a higher signal to noise ratio (*I*/σ >9) and a resolution up to 2.2 Å (Table 1). Despite the weak diffraction data statistics, we proceeded to the structure analysis, taking advantage of the consistent folding of hemoglobin. The phase was recovered using the molecular replacement method with lowland deer mouse hemoglobin as the search model (PDB ID: 4H2L) in Phenix [24]. The obtained solution was manually fitted to the electron density using Coot [25] and was then further refined by running phenix.refine. The fitting between the electron density map and the model was good. However, the last residue in all of the α chains, α141Arg, was not assigned in the final model because of weak electron density. The crystallographic statistics are listed in Table 1. The structure was deposited in the Protein Data Bank with the ID code 5KER. The obtained highland hemoglobin structure had two heterotetrameric hemoglobin molecules in the asymmetric unit. The chain ID allocations in this PDB entry were as follows: one tetramer consisted of chains A (α_1_), B (β_1_), C (α_2_) and D (β_2_), and the other of chains E, F, G and H in the same order. PyMOL (Version 1.8 Schrödinger, LLC.) was used for structural mining.

## Results

### Structure of the highland hemoglobin variant

The type S highland hemoglobin crystal contained 2 tetramers in the asymmetric unit (Fig 1B) that were connected by intersubunit hydrogen bonds between subunits C, D, E and F (Table 2). Although the UV-visible spectrum of the dissolved crystal solution indicated the presence of carboxyhemoglobin, we were only able to assign a water molecule at each heme based on the obtained electron density map. In addition, chain H (β subunit), did not have an assigned ligand, as there was no electron density supporting the presence of a molecule of carbon monoxide, oxygen, or water (S2 Fig). Lowland hemoglobin was also in water-bound form, but it had one α and one β subunit in the asymmetric unit [20], as is often observed in liganded tetrameric hemoglobin structures including human hemoglobin [26].

**Table 2:** Interacting amino acids of the 2 tetramers.

Subunit interaction (area)	Interaction	Distance
Chain C/E (113.2 Å^2^)	α_1_C-Lys16 (NZ):α_1_E-Ala111 (O)	3.21 Å
Chain C/F (232.2 Å^2^)	α_1_C-His20 (NE2):β_1_F-His117 (O)	3.56 Å
	α_1_C-Ala115 (N):β_1_F-Asp121 (OD1)	3.07 Å
Chain D/F (56.1 Å^2^)	β_2_D-Lys120 (NZ):β_1_F-Gly16 (O)	2.32 Å

At the tetramer interface, chain F and C had the highest subunit interaction area. One of the identified hydrogen bonds between α_1_C-Ala115 (N) and β_1_F-Asp121 (OD1) could be unique, as the lowland hemoglobin variant has Ser at α115. However, none of the identified interactions was located near the distal heme pocket. The two tetramers folded similarly with an r.m.s. value of 0.54 between ABCD and EFGH according to the PyMOL alignment function. Of note, in the sickle-cell deoxyhemoglobin (deoxyHbS) fiber using the same chain designation as the present structure, the head to tail tetramer interactions were α_1_C-Pro114: β_1_F-Glu121 [27]. The interactions observed during polymerization were different between deoxyHbS and the present structure.

Main chain traces between each subunit of the highland and lowland hemoglobin variants were highly similar, with r.m.s.d. values falling within 0.3–0.8 Å, except for the terminal residues using the CCP4 superposing program [28]. Higher displacements in the main chain atoms were found in the α subunit residues between 41 and 54 with r.m.s.d. values of 1.2–2.6 Å. This was caused by the amino acid mutation at position α50 (Fig 1C; see the following section). β subunit main chain traces between highland and lowland hemoglobin showed no detectable displacements.

### Effects of the α50 mutation on the configuration of αHis45 and the α_1_β_2_/α_2_β_1_ switch interface

Among the 12 amino acid mutations that distinguish the lowland and highland hemoglobin variants, one especially noteworthy change involved the replacement of αPro50 in highland hemoglobin for αHis50 in lowland hemoglobin (Fig 1A and 1C). Based on the main chain atom displacements, this αHis50Pro mutation changed the coordinates of the nearby loop region as well as that of αHis45. The α50 and αHis45 residues are located in the loop region leading to the E helix, and this loop is relatively rigid because it is stabilized by hydrogen bonds according to the Dictionary of Secondary Structure of Proteins (DSSP) [29, 30] (data not shown). In the lowland hemoglobin variant, αHis50 interacts with αGlu30 (Fig 1C, [19]), which pinches the amino end of the loop and prevents a stable αHis45-heme interaction. This resulted in αHis45 interacting with nearby residues in the crystal symmetry relationship [20]. In the highland hemoglobin variant, this pinching was not observed because αPro50 has no interaction with αGlu30. As a result, the highland hemoglobin variant could have a stable αHis45-heme interaction. In fish hydrated hemoglobin, the contribution of αHis45 in heme retention reported [31], which supports our assumptions.

**Fig 2:**
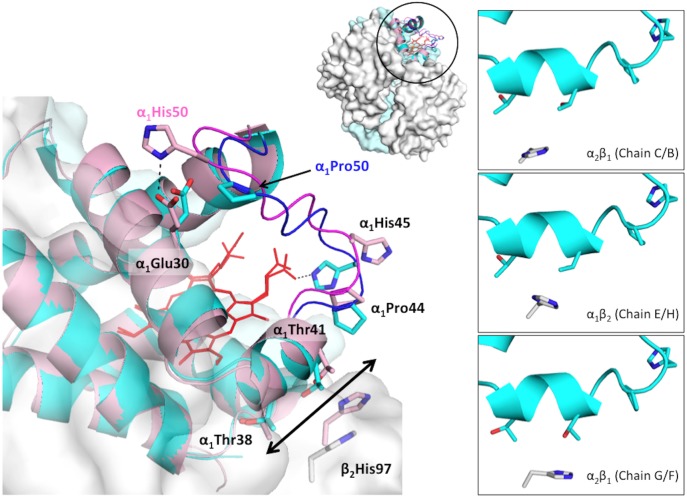
Difference in the α_1_β_2_ switching region interaction between highland and lowland hemoglobin. The α subunit is shown in the cartoon, and the key residues are represented as a stick model. The residues unique to highland and lowland hemoglobin are labeled in cyan and pink, respectively. The highland hemoglobin α_1_ subunit is colored in cyan, and its β_2_ subunit is colored in gray. Both subunits from the lowland hemoglobin are colored in pink. The right side boxes indicate differences in the α_1_β_2_/α_2_β_1_ interface coordinates, particularly the β_2_His 97 imidazole ring orientation relative to the α_1_ residues.

The difference in the loop region also affected intersubunit interactions. Hemoglobin exhibits allosteric motion in the transition between the T and R states, and this oxygenation-linked transition alters a set of subunit contacts at the α_1_β_2_/α_2_β_1_ switching region (Fig 2). In human hemoglobin, the highlighted residues at this switching region are Thr38, Thr41 and Pro44 from the α_1_/α_2_ subunit C/D helix and His97 from the β_2_/β_1_ subunit [32, 33]. In deoxygenated hemoglobin (T), β_2_His97 is located close to α_1_Pro44, whereas in oxygenated hemoglobin (R), β_2_His97 faces away from α_1_Pro44 [10, 32, 33]. In the case of deer mouse hemoglobin (Fig 1A), both highland and lowland hemoglobin variants have the same amino acid residues at the switch region as the case of human hemoglobin. Although the superimposed structure showed that the distance between α_1_Thr38 C_α_ from highland and lowland hemoglobin was within a 0.3 Å difference, the distance comparisons between the α_1_Thr41 pairs and α_1_Pro44 pairs increased to 1.1 Å and 1.4 Å, respectively (Fig 2). In addition, the distance between α_1_Thr38, α_1_Thr41 and α_1_Pro44 with respect to β_2_His97 was shorter in the lowland hemoglobin variant than in the highland variant (Table 3).

**Table 3:** C_α_ distances at α_1_β_2_/α_2_β_1_ switching region.

Hemoglobin	Interface (chains)	α_1_Thr38:β_2_His 97	α_1_Thr41:β_2_His 97	α_1_Pro44:β_2_His 97
Lowland	α_1_β_2_ (A/D)	5.0 Å	7.3 Å	12.2 Å
Highland	α_1_β_2_ (A/D)	6.7 Å	8.7 Å	13.9 Å
	α_2_β_1_ (C/B)	6.7 Å	8.7 Å	13.7 Å
	α_1_β_2_ (E/H)	6.0 Å	7.7 Å	12.9 Å
	α_2_β_1_ (G/F)	6.5 Å	8.3 Å	13.3 Å

Although the C_α_ coordinates of residues at each switching region from highland hemoglobin were the same, the relative orientation of the β_2_His97 imidazole ring was different, particularly at chain G and F (α_2_β_1_ interface, Fig 2).

**Fig 3:**
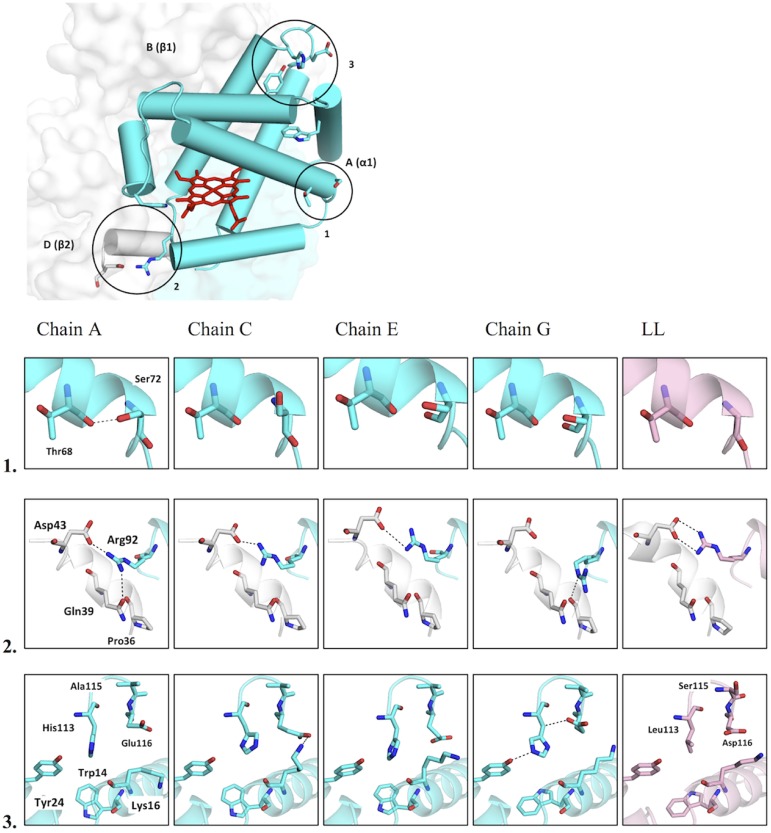
Unique interactions present in highland hemoglobin. Locations of unique interactions. Chain A (also referred as α1) is colored in cyan, and chain B (β1) and chain D (β2) are colored in white. The key residues are shown as a stick model, and the circles highlight the locations of identified unique interactions.

Comparisons of subunit interfaces between the highland and lowland hemoglobin variants using the PISA webserver [34] showed other differences in the α_1_β_2_/α_2_β_1_ interface interactions. For example, the interaction between α_1_Arg92 and β_2_Asp43 was different due to the change in the orientation of α_1_Arg92. This particular interaction was not observed between chains G (α subunit) and F (β subunit) (Fig 3) in highland hemoglobin. Nevertheless, the α_1_Arg92 / β_2_Asp43 interaction was found in chain H, the subunit without assigned ligand (Fig 1B and S2 Fig) Since chain H had a planar heme like a typical in liganded hemoglobin, alterations of α_1_β_2_/α_2_β_1_ interface appear to affect hemoglobin-oxygen affinity.

**Fig 4:**
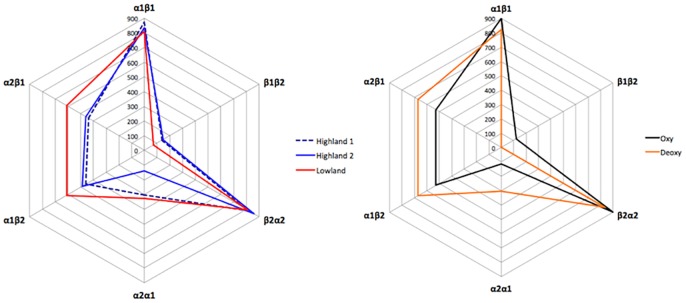
Difference in interface area between highland and lowland hemoglobin and human oxygenated and deoxygenated hemoglobin. Interface area of lowland hemoglobin and two highland hemoglobin tetramers were calculated by the PISA webserver. The values on the graph show the surface area in Å^2^. The blue line is a result from one highland hemoglobin, which consists of chains A, B, C and D. The dotted dark line is a result from another highland hemoglobin (chains E, F, G and H). The lowland hemoglobin result is depicted by the red line. The intradimeric interface area of human hemoglobin was calculated using the following structures; deoxygenated structure (2DN2, orange) and oxygenated structure (2DN1, black)

In addition, highland hemoglobin has a reduced subunit contact surface at the α_1_β_2_/α_2_β_1_ interface, and this difference in interface area between the lowland and highland hemoglobin variants is similar in magnitude to the difference between oxygenated and deoxygenated states of human hemoglobin (Fig 4). In particular, lowland hemoglobin has about 30% larger α_1_β_2_/α_2_β_1_ interface that is expected for the T-state [20]. Just as the T-state quaternary structure has lower oxygen-binding affinity than the R-state quaternary structure [35, 36], the highland hemoglobin variant has a higher oxygen-binding affinity than lowland hemoglobin variant.

### α_1_β_1_ interface changes induced by amino acid mutations

Previous structural analysis of the lowland hemoglobin variant suggested that the α_1_β_1_ interface interaction (in particular, the interaction between α_1_Cys34 and β_1_Ser128) differs from that of the highland variant due to a mutation at β_1_Ala128 [20]. Our structural analysis revealed that the highland hemoglobin variant lacked this interaction, and also showed that the α_1_Cys34 side chain was pointed toward the subunit interface, allowing the interaction with the β_1_Pro124 main chain oxygen atom (S3 Fig).

Mutations at α_1_113, α_1_115 and α_1_116 might also affect this interface as the β_1_128 mutation. The lowland hemoglobin variant has α_1_Leu113, α_1_Ser115 and α_1_Asp116, whereas the highland hemoglobin has α_1_His113, α_1_Ala115 and α_1_Glu116. Structural analysis of the highland hemoglobin variant demonstrated that α_1_His113 and α_1_Glu116 had different coordinates among the four α subunits (Fig 3-3), resulting in different interactions with nearby residues. For example, α_1_Glu116 from chain C showed an interaction with α_1_Lys16, and chain G α_1_His113 interacted with α_1_Tyr24 and α_1_Glu116. In the lowland hemoglobin variant, these residues show no interactions with nearby residues. In addition, α_1_Trp14, which alters conformations between the deoxygenated and oxygenated states in human hemoglobin [37], seems to have a different set of interactions in deer mouse hemoglobin. In lowland hemoglobin, the α_1_Trp14 side chain was oriented toward residues α_1_113, α_1_115 and α_1_116, whereas the majority of the highland hemoglobin had an α_1_Trp14 side chain pointed toward α_1_Tyr24 (Fig 3-3). Only chain G from the highland hemoglobin had the same α_1_Trp14 coordinate as the lowland hemoglobin. Because chain G had the highest number of interactions between residues at α_1_113, α_1_115 and α_1_116, mutations at these sites can be expected to affect the packing of nearby helices in the α subunit. Such changes could account for differences in the nature of atomic contacts at the α_1_β_1_ interface.

## Discussion

The hemoglobin variants of highland and lowland deer mice are distinguished by 12 amino acid mutations. We compared structures of both variants to elucidate how these mutations account for the observed difference in hemoglobin-oxygen affinity. A previous study of the lowland hemoglobin variant revealed no atomic contact between αTrp14 and E helix residue α67, which enhances the mobility of the E-helix (which contains the distal histidine, αHis58) [20]. In the lowland hemoglobin variant, the interaction between αHis50 and αGlu30 might promote tight α subunit packing as well as helix mobility [19]. In addition, the evidence suggests that the α_1_Cys34-β_1_Ser128 interaction in the lowland hemoglobin variant produced a change in the α_1_β_1_ interface [19, 20]. Three mutations at α113, α115 and α116 highlighted changes in nearby helix packing that stem from changes in inter-helix interactions. Additionally, αSer71 in the highland variant interacts with αThr68; an interaction that is not possible in the lowland variant due to the presence of αAla71 (Fig 3-1).

The α_1_β_2_/α_2_β_1_ interfaces were different because of the main chain atom displacements between α41 and α54 and the stability of the αHis45-heme interaction, and these were enhanced by the αHis/Pro50 mutation. Additionally, the α_1_β_1_ interface was altered, as the highland hemoglobin β_1_Ala128 substitution prevented interaction with α_1_Cys34, and this promoted an interaction between α_1_Cys34 and the β_1_Pro124 main chain oxygen atom.

In conclusion, the amino acid mutations that distinguish the highland and lowland deer mouse hemoglobin variants affect oxygen-binding affinity by altering interface interactions, which are mainly attributable to mutations in the α subunit. The highland hemoglobin variant has a looser α_1_β_2_/α_2_β_1_ switch region than the lowland variant due to the α50 mutation, and the nature of atomic contacts at the α_1_β_1_ interface is also different because of mutations at β128, α113, α115 and α116. In guinea pig hemoglobin [38], the lack of a salt bridge between αThr30 and αPro50 conferred an increased flexibility. Also, the guinea pig hemoglobin has αHis40 instead of αPro40 (as in human hemoglobin), and the bulkiness of histidine affects the α_1_β_2_/α_2_β_1_ interface during the T↔R transition in quaternary structure. These results suggest that the modulation of allosteric interactions via changes in protein flexibility may represent an important mechanism in the evolutionary fine-tuning of hemoglobin-oxygen affinity.

## Supporting information

S1 Fig Pictures of highland hemoglobin crystals. (A) Schematic figure of the setting hemoglobin crystal. The highland hemoglobin drop was placed in the airtight glass vial coated with silicon. After injecting the reservoir, carbon monoxide was purged from the vial. (B) Picture of the crystallization system described in Fig 1A. (C) Type F crystals in the cryo-loop during data collection. (D) Type S crystals in a droplet. The white arrow indicates the location of the crystal used for the data collection. In C and D, white bars indicate 0.1 mm.
(TIF)

S2 Fig The electron density map around heme in chain H. Comparisons of the 2*F*_*o*_-*F*_*c*_ map around β subunit hemes between chain H (no ligand bound) and chain B (water bound shown as a red sphere). The contour level is 1.5 sigma, and bound water molecule is shown in red sphere (figure was generated by PyMOL).
(TIF)

S3 Fig Differences in α_1_β_1_ interface due to amino acid substitution. The highland hemoglobin α subunit is colored in cyan, and the lowland hemoglobin α subunit is colored in pink. The corresponding β subunit is colored in gray. Key residues at the α_1_β_1_ interface are labeled, and interactions identified by the PISA webserver are depicted with black dotted lines.
(TIF)

## References

[1] Perutz MF. Structure of hemoglobin. Brookhaven symposia in biology. 1960;13:165–83. 13734651

[2] Perutz MF. Stereochemistry of cooperative effects in haemoglobin. Nature. 1970;228(5273):726–39. Epub 1970/11/21. 5528785 10.1038/228726a0

[3] Perutz MF. Nature of haem-haem interaction. Nature. 1972;237(5357):495–9. 12635193 10.1038/237495a0

[4] Birukou I, Soman J, Olson JS. Blocking the gate to ligand entry in human hemoglobin. The Journal of biological chemistry. 2011;286(12):10515–29. PubMed Central PMCID: PMCPMC3060505. 10.1074/jbc.M110.176271 21193395 PMC3060505

[5] Shadrina MS, English AM, Peslherbe GH. Effective simulations of gas diffusion through kinetically accessible tunnels in multisubunit proteins: O2 pathways and escape routes in T-state deoxyhemoglobin. Journal of the American Chemical Society. 2012;134(27):11177–84. 10.1021/ja300903c 22690872

[6] Srajer V, Ren Z, Teng TY, Schmidt M, Ursby T, Bourgeois D, et al Protein conformational relaxation and ligand migration in myoglobin: a nanosecond to millisecond molecular movie from time-resolved Laue X-ray diffraction. Biochemistry. 2001;40(46):13802–15. 11705369 10.1021/bi010715u

[7] Srajer V, Royer WE Jr. Time-resolved x-ray crystallography of heme proteins. Methods in enzymology. 2008;437:379–95. PubMed Central PMCID: PMC3287071. 10.1016/S0076-6879(07)37019-5 18433638 PMC3287071

[8] Srajer V, Teng T, Ursby T, Pradervand C, Ren Z, Adachi S, et al Photolysis of the carbon monoxide complex of myoglobin: nanosecond time-resolved crystallography. Science. 1996;274(5293):1726–9. 8939867 10.1126/science.274.5293.1726

[9] Rubin MM, Changeux JP. On the nature of allosteric transitions: implications of non-exclusive ligand binding. Journal of molecular biology. 1966;21(2):265–74. 5972463 10.1016/0022-2836(66)90097-0

[10] Fischer S, Olsen KW, Nam K, Karplus M. Unsuspected pathway of the allosteric transition in hemoglobin. Proceedings of the National Academy of Sciences of the United States of America. 2011;108(14):5608–13. PubMed Central PMCID: PMC3078355. 10.1073/pnas.1011995108 21415366 PMC3078355

[11] Adachi S, Park SY, Tame JRH, Shiro Y, Shibayama N. Direct observation of photolysis-induced tertiary structural changes in hemoglobin. Proceedings of the National Academy of Sciences of the United States of America. 2003;100(12):7039–44. 10.1073/pnas.1230629100 12773618 PMC165826

[12] Storz JF, Scott GR, Cheviron ZA. Phenotypic plasticity and genetic adaptation to high-altitude hypoxia in vertebrates. J Exp Biol. 2010;213(Pt 24):4125–36. PubMed Central PMCID: PMCPMC2992463. 10.1242/jeb.048181 21112992 PMC2992463

[13] Storz JF. Hemoglobin–oxygen affinity in high-altitude vertebrates: is there evidence for an adaptive trend? The Journal of experimental biology. 2016;219(20):3190–203. 27802149 10.1242/jeb.127134 PMC5091379

[14] Storz JF, Moriyama H. Mechanisms of hemoglobin adaptation to high altitude hypoxia. High Alt Med Biol. 2008;9(2):148–57. Epub 2008/06/27. 10.1089/ham.2007.1079 18578646 PMC3140315

[15] Jensen B, Storz JF, Fago A. Bohr effect and temperature sensitivity of hemoglobins from highland and lowland deer mice. Comp Biochem Physiol A Mol Integr Physiol. 2016;195:10–4. PubMed Central PMCID: PMCPMC4789091. 10.1016/j.cbpa.2016.01.018 26808972 PMC4789091

[16] Storz JF, Runck AM, Sabatino SJ, Kelly JK, Ferrand N, Moriyama H, et al Evolutionary and functional insights into the mechanism underlying high-altitude adaptation of deer mouse hemoglobin. Proceedings of the National Academy of Sciences of the United States of America. 2009;106(34):14450–5. Epub 2009/08/12. 10.1073/pnas.0905224106 19667207 PMC2732835

[17] Natarajan C, Hoffmann FG, Lanier HC, Wolf CJ, Cheviron ZA, Spangler ML, et al Intraspecific polymorphism, interspecific divergence, and the origins of function-altering mutations in deer mouse hemoglobin. Mol Biol Evol. 2015;32(4):978–97. PubMed Central PMCID: PMCPMC4379404. 10.1093/molbev/msu403 25556236 PMC4379404

[18] Storz JF, Runck AM, Moriyama H, Weber RE, Fago A. Genetic differences in hemoglobin function between highland and lowland deer mice. J Exp Biol. 2010;213(Pt 15):2565–74. Epub 2010/07/20. 10.1242/jeb.042598 20639417 PMC2905302

[19] Natarajan C, Inoguchi N, Weber RE, Fago A, Moriyama H, Storz JF. Epistasis among adaptive mutations in deer mouse hemoglobin. Science. 2013;340(6138):1324–7. PubMed Central PMCID: PMC4409680. 10.1126/science.1236862 23766324 PMC4409680

[20] Inoguchi N, Oshlo JR, Natarajan C, Weber RE, Fago A, Storz JF, et al Deer mouse hemoglobin exhibits a lowered oxygen affinity owing to mobility of the E helix. Acta Crystallogr Sect F Struct Biol Cryst Commun. 2013;69(Pt 4):393–8. PubMed Central PMCID: PMCPMC3614163. 10.1107/S1744309113005708 23545644 PMC3614163

[21] Yokoyama T, Neya S, Tsuneshige A, Yonetani T, Park SY, Tame JR. R-state haemoglobin with low oxygen affinity: crystal structures of deoxy human and carbonmonoxy horse haemoglobin bound to the effector molecule L35. Journal of molecular biology. 2006;356(3):790–801. 10.1016/j.jmb.2005.12.018 16403522

[22] Natarajan C, Jiang X, Fago A, Weber RE, Moriyama H, Storz JF. Expression and purification of recombinant hemoglobin in Escherichia coli. PLoS One. 2011;6(5):e20176 PubMed Central PMCID: PMC3098879. 10.1371/journal.pone.0020176 21625463 PMC3098879

[23] Otwinowski Z, Minor W. Processing of X-ray Diffraction Data Collected in Oscillation Mode In: Carter JCWS, R M, editor. Methods in Enzymology 276. 276 New York: Academic Press; 1997 p. 307–26. 10.1016/S0076-6879(97)76066-X 27754618

[24] Adams PD, Afonine PV, Bunkoczi G, Chen VB, Davis IW, Echols N, et al PHENIX: a comprehensive Python-based system for macromolecular structure solution. Acta Crystallogr D Biol Crystallogr. 2010;66(Pt 2):213–21. PubMed Central PMCID: PMCPMC2815670. 10.1107/S0907444909052925 20124702 PMC2815670

[25] Emsley P, Lohkamp B, Scott WG, Cowtan K. Features and development of Coot. Acta Crystallogr D Biol Crystallogr. 2010;66(Pt 4):486–501. PubMed Central PMCID: PMCPMC2852313. 10.1107/S0907444910007493 20383002 PMC2852313

[26] Park SY, Yokoyama T, Shibayama N, Shiro Y, Tame JR. 1.25 A resolution crystal structures of human haemoglobin in the oxy, deoxy and carbonmonoxy forms. Journal of molecular biology. 2006;360(3):690–701. Epub 2006/06/13. 10.1016/j.jmb.2006.05.036 16765986

[27] Wishner BC, Ward KB, Lattman EE, Love WE. Crystal structure of sickle-cell deoxyhemoglobin at 5 A resolution. Journal of molecular biology. 1975;98(1):179–94. Epub 1975/10/15. 1195378 10.1016/s0022-2836(75)80108-2

[28] Kabsch W. A solution for the best rotation to relate two sets of vectors. Acta Crystallographica Section A. 1976;32:922–3.

[29] Joosten RP, te Beek TA, Krieger E, Hekkelman ML, Hooft RW, Schneider R, et al A series of PDB related databases for everyday needs. Nucleic Acids Res. 2011;39(Database issue):D411–9. PubMed Central PMCID: PMCPMC3013697. 10.1093/nar/gkq1105 21071423 PMC3013697

[30] Kabsch W, Sander C. Dictionary of protein secondary structure: pattern recognition of hydrogen-bonded and geometrical features. Biopolymers. 1983;22(12):2577–637. 10.1002/bip.360221211 6667333

[31] Aranda Rt, Cai H, Worley CE, Levin EJ, Li R, Olson JS, et al Structural analysis of fish versus mammalian hemoglobins: effect of the heme pocket environment on autooxidation and hemin loss. Proteins. 2009;75(1):217–30. PubMed Central PMCID: PMCPMC2649966. 10.1002/prot.22236 18831041 PMC2649966

[32] Voet D, Voet JG. Biochemistry. 4th ed. Hoboken, NJ: John Wiley & Sons; 2011 xxv, 1428, I-53 p. p.

[33] Baldwin J, Chothia C. Haemoglobin: the structural changes related to ligand binding and its allosteric mechanism. Journal of molecular biology. 1979;129(2):175–220. 39173 10.1016/0022-2836(79)90277-8

[34] Krissinel E, Henrick K. Inference of macromolecular assemblies from crystalline state. Journal of molecular biology. 2007;372(3):774–97. 10.1016/j.jmb.2007.05.022 17681537

[35] Viappiani C, Abbruzzetti S, Ronda L, Bettati S, Henry ER, Mozzarelli A, et al Experimental basis for a new allosteric model for multisubunit proteins. Proceedings of the National Academy of Sciences of the United States of America. 2014;111(35):12758–63. PubMed Central PMCID: PMCPMC4156698. 10.1073/pnas.1413566111 25139985 PMC4156698

[36] Di Cera E, Robert CH, Gill SJ. Allosteric interpretation of the oxygen-binding reaction of human hemoglobin tetramers. Biochemistry. 1987;26(13):4003–8. 3651431 10.1021/bi00387a039

[37] Balakrishnan G, Tsai CH, Wu Q, Case MA, Pevsner A, McLendon GL, et al Hemoglobin site-mutants reveal dynamical role of interhelical H-bonds in the allosteric pathway: time-resolved UV resonance Raman evidence for intra-dimer coupling. Journal of molecular biology. 2004;340(4):857–68. 10.1016/j.jmb.2004.05.013 15223326

[38] Pairet B, Jaenicke E. Structure of the altitude adapted hemoglobin of guinea pig in the R2-state. PloS one. 2010;5(8):e12389 PubMed Central PMCID: PMCPMC2927554. 10.1371/journal.pone.0012389 20811494 PMC2927554

