# Supplementary figures and images for: Alteration of the α1β2/α2β1 subunit interface contributes to the increased hemoglobin-oxygen affinity of high-altitude deer mice
Source: PLoS One. 2017 Mar 31;12(3):e0174921. doi: 10.1371/journal.pone.0174921 (PMC5376325; doi:10.1371/journal.pone.0174921)

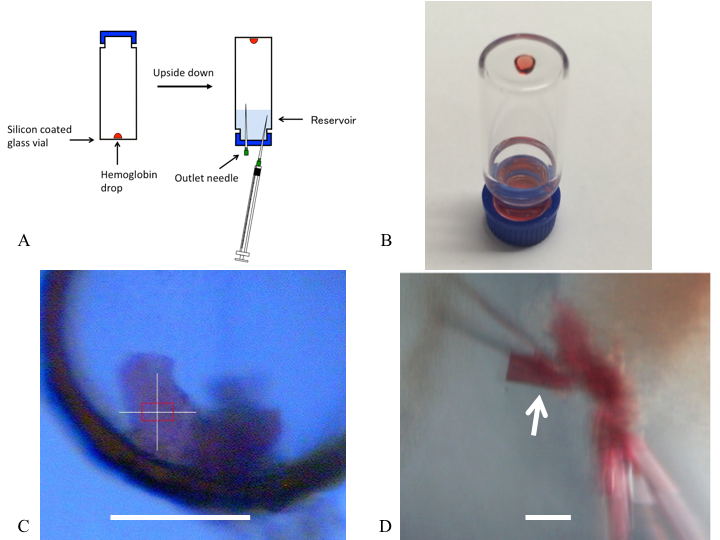

Supplement: S1 Fig — (A) Schematic figure of the setting hemoglobin crystal. The highland hemoglobin drop was placed in the airtight glass vial coated with silicon. After injecting the reservoir, carbon monoxide was purged from the vial. (B) Picture of the crystallization system described in Fig 1A. (C) Type F crystals in the cryo-loop during data collection. (D) Type S crystals in a droplet. The white arrow indicates the location of the crystal used for the data collection. In C and D, white bars indicate 0.1 mm. (TIF) [file pone.0174921.s001.tif]

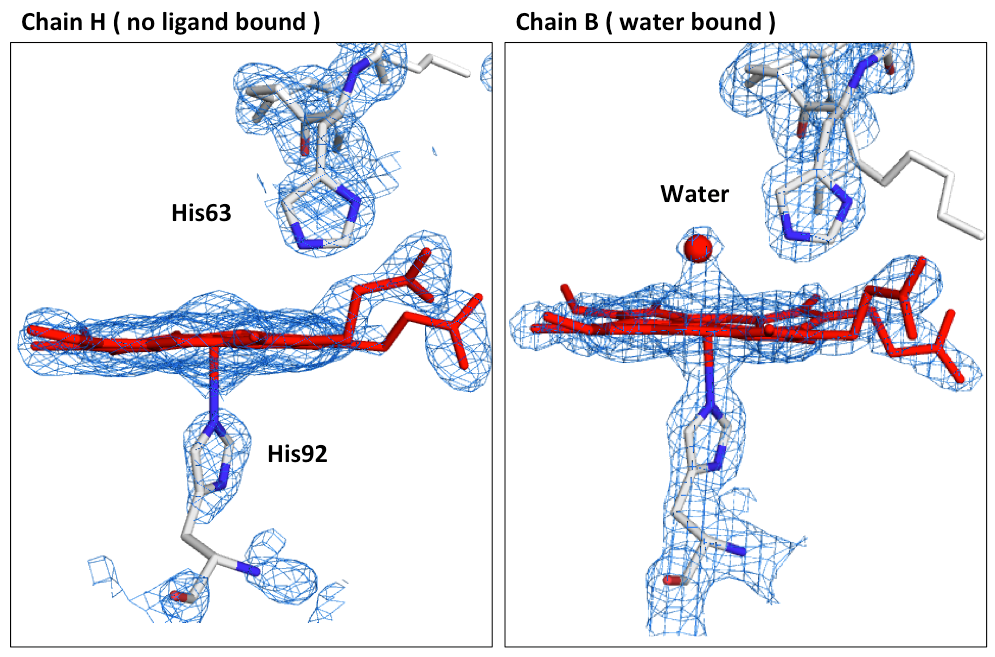

Supplement: S2 Fig — Comparisons of the 2Fo-Fc map around β subunit hemes between chain H (no ligand bound) and chain B (water bound shown as a red sphere). The contour level is 1.5 sigma, and bound water molecule is shown in red sphere (figure was generated by PyMOL). (TIF) [file pone.0174921.s002.tif]

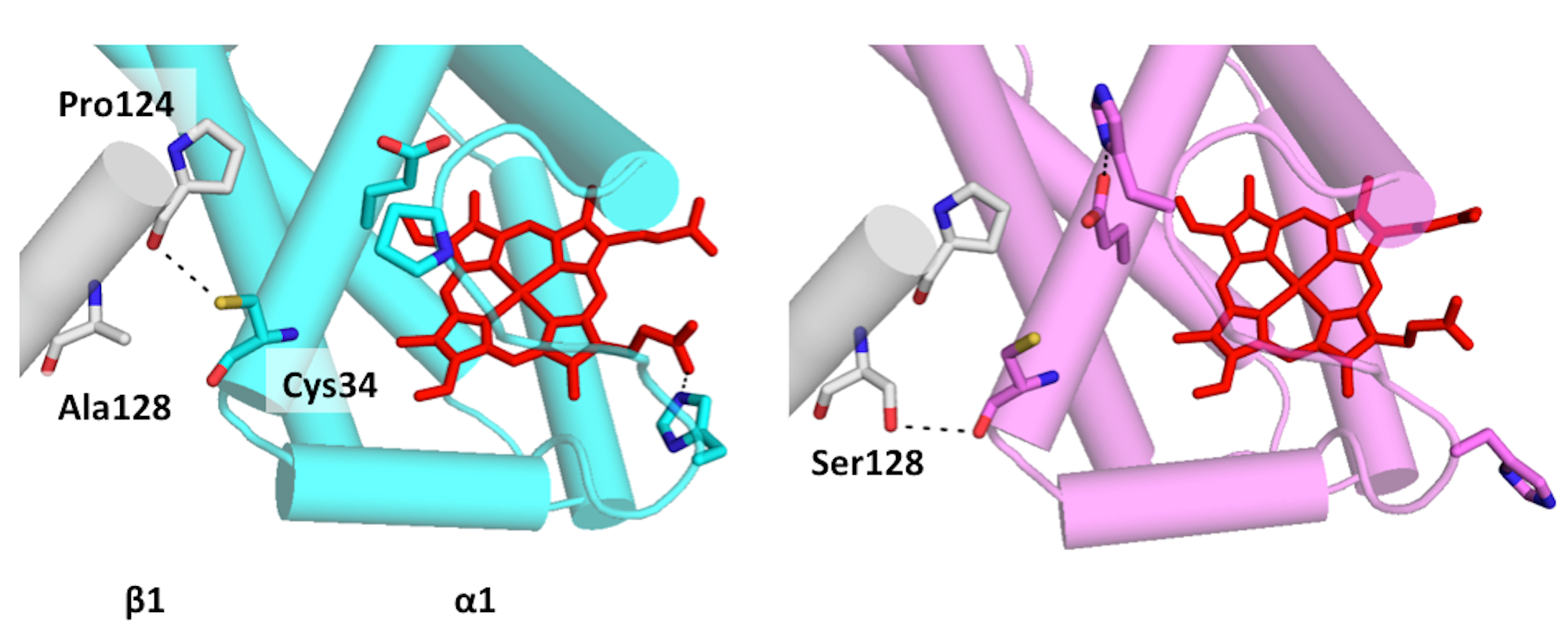

Supplement: S3 Fig — The highland hemoglobin α subunit is colored in cyan, and the lowland hemoglobin α subunit is colored in pink. The corresponding β subunit is colored in gray. Key residues at the α1β1 interface are labeled, and interactions identified by the PISA webserver are depicted with black dotted lines. (TIF) [file pone.0174921.s003.tif]
